# Assessment of Epidemiological Indicators for Evaluation of National Malaria Elimination Programme: A Retrospective Study

**DOI:** 10.4314/ejhs.v33i6.12

**Published:** 2023-11

**Authors:** Hima Sree Polisetti, K R Vinay Rajan, K Eswar Kumar

**Affiliations:** 1 M. Pharm-Pharmacy Practice, A.U. College of Pharmaceutical Sciences, Andhra University, Visakhapatnam, 530003; 2 A.U. College of Pharmaceutical Sciences, Andhra University, Visakhapatnam. 530003

**Keywords:** Malaria, National Malaria Elimination Programme, National Framework for Malaria Elimination in India, Annual Parasite Incidence, Plasmodium falciparum

## Abstract

**Background:**

Malaria has been one of India's most considerable health problems since 1940. The objective of our study is to determine the status of the National Malaria Elimination Programme in India by using epidemiological indicators.

**Methods and Materials:**

The annual reports of malaria for the years 2014-2021 and monthly reports for 2020 and 2021 were collected from the official web portal and were analysed for study specific assessments.

**Results:**

The API has shown a statistically significant reduction from 2017-2021 in all states along with category-1(P=0.003) and category-2(P=0.029) states/UTs, but there was no statistically significant reduction from 2017-2021 in category-3 (P=0.166) states/UTs. The zero indigenous cases had not been achieved in category-1 states/UTs. The overall percentage reduction in number of malaria cases in 2020 at the national level compared with 2014 was 83.6%. Despite states with strong health systems such as Gujarat, Maharashtra and Karnataka, have not shown zero indigenous cases in 2020 and the malaria cases noted were very far from reaching the targets.

**Conclusions:**

Although we observed a significant drop in malaria incidence from 2014 to 2020, demonstrating that the country is moving nearer to malaria elimination, it is crucial to implement the strategies to reduce Plasmodium falciparum% and re-establish surveillance programmes and execute national and state programmes in order to achieve the success of the National Malaria Elimination Programme. The recategorization of states/UTs are in accordance to the API, and implementation strategies were also needed.

## Introduction

India is the largest contributor of total malaria cases with an account of 79% of amongst the WHO South-East Asia Region, and most of the cases are due to the *P. vivax* (1). Malaria has been one of India's most considerable health problems since 1940. Controlled interruption of a specific malaria parasite species transmission in a particular geographic region is described as eliminating malaria (2). Till now, WHO has granted malaria-free certification to 40 countries and territories globally. Since, China, as one of the world's most populous nations, managed to attain malaria-free certification in 2021, it set an example for India that eliminating malaria is an achievable goal. (3).

Although the first National Malaria Control Programme (NMCP) was launched in 1953, the complete elimination of malaria in India has still not been attained due to many reasons, such as the transmission and overlap of multiple *Plasmodium* species and *Anopheles* vectors, insecticide resistance, and antimalarial drug resistance, and impact of climate change on the above factors (4,5).

Presently, malaria in India is under the National Vector Borne Disease Control (NVBDC), which was launched in 2002 by combining three ongoing programmes, such as malaria (National Anti -Malaria Control Programme), filaria (Filaria Control Programme) and kala azar (Kala Azar Control programme) (6). It is a part of the Technical Division of the Directorate General of Health Services, Government of India, for the control of 6 vector borne diseases; namely, malaria, filariasis, kala azar, Japanese encephalitis, chikungunya, and dengue (6).

The National Centre for Vector Borne Disease Control (NCVBDC) of the Ministry of Health and Family Welfare officially unveiled the National Framework for Malaria Elimination (NFME) in India (2016–2030) on February 11, 2016, with the vision of eliminating malaria nationally and contributing to improved health, quality of life, and alleviation of poverty (7). Aligning with the vision of NFME, the National Strategic Plan 2017-2022, focuses on strategic policies to provide universal intervention package, paving the way for malaria elimination by 2030 (8).

As a part of this framework, the states/UTs were subdivided into four categories with Annual Parasite Incidence (API) as a primary criterion such as Category 0-zero indigenous cases of malaria, Category 1-States/UTs including their districts reporting an API of less than 1 case per 1000 population at risk, Category 2-States/UTs with an API of less than 1 case per 1000 population at risk, but some of their districts still reporting an API of 1 case per 1000 population at risk or above, Category 3-States/UTs with an API of 1 case per 1000 population at risk or above (7).

The NFME has setup several milestones and targets to be reached by the end of 2020, 2022, 2024, 2027 and 2030, such as an estimated reduction of 15-20% of malaria cases at the national level compared to 2014 in 2020, all states in category-1 have to achieve zero indigenous cases by 2020. States with strong healthcare systems have to achieve zero indigenous cases by 2020, zero indigenous cases in all category-2 states/UTs by 2022. Transmission of malaria interrupted and zero indigenous cases and deaths due to malaria shall be attained in all 31 states/UTs by 2024. In addition, the indigenous transmission of malaria in India should be interrupted by 2027, and the malaria-free status shall be maintained throughout the nation by 2030 (7). As 2020 has been completed, there is an urge to evaluate the performance of the National Frame work and its strategies for achieving the milestones laid down under the framework. Hence, our present study was planned to determine the progress and success of the NFME.

## Methods

This study qualifies for the exception of ethics committee permission as this is secondary research and no informed consent process was involved, because the data was collected, synthesized, and analysed from the open-source public domain i.e., NCVBDC.

**Figure 1:**
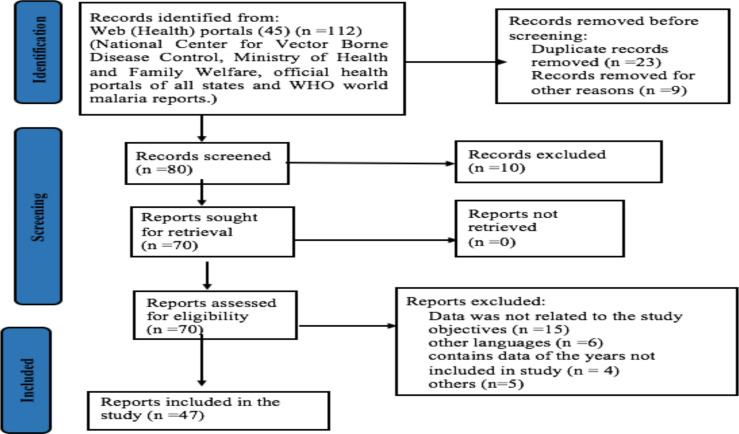
Study flow chart

The official web portals of various states and the central government were searched for malaria reports including the NCVBDC, the National Institute of Malaria Research (NIMR), and the Ministry of Health and Family Welfare. Included only English reports; excluded other languages. Accessed National Framework for Malaria Elimination. Collected reports from the National Center for Vector Borne Diseases Control, such as Malaria annual reports (2017-2018), Monthly malaria situation reports (2020 January-2021 December) for category-1, -2, -3 states/UTs, Trends of malaria parameters in India (2017-2021) (9), Malarial situation in India reports (2014, 2018)(10) and Annual Report 2014-15(11). Excluded reports from NIMR and other portals not aligned with study objectives (Figure 1).

The primary objective of our study is to determine the status of the NFME in India by using epidemiological indicators. The secondary objectives include (i) Transmission of malaria interrupted and zero indigenous cases and deaths in Category-1 states/UTs in 2020 (ii) All 11 states/UTs under Category 2 in 2014 entered into Category 1 in 2020, (iii) Five states/UTs under Category 3 in 2014 which entered into Category 2 i 2020 (iv) Five states/UTs under Category 3 (intensified control phase) in 2014 that reduced malaria transmission, but continued to remain in Category 3 in 2020. (v) an estimated reduction in the malaria of 15–20% by 2020 at the national level compared with 2014, (vi) additionally, progressive states with strong health systems such as Gujarat, Maharashtra and Karnataka may implement accelerated malaria elimination programmes to achieve interruption of transmission and demonstrate early elimination followed by the sustenance of zero indigenous cases by 2020.

The study's assessment indicators include the Number of malaria cases, Number of *Plasmodium falciparum* cases, Annual Blood Smear Examination Rate (ABER), Annual Parasite Incidence (API), Annual Falciparum Incidence (AFI), Slide Positivity rate (SPR), Slide falciparum Rate (SFR), and *Plasmodium falciparum* Percentage (Pf %) (12).

The data from 2014 to 2021of malaria was collected from the above-mentioned reports in MS Excel 2019. The study's assessment parameters were calculated for 2014,2017-2020 from the above-collected data in Ms Excel. Statistical analysis, ANOVA and Chi-square test was performed with Epi Info 7.2 (Center for Disease Control and Prevention, USA), Paired t-test was performed in GraphPad Prism 8.0.1.

## Results

The retrospective analysis of malaria in India (by analysis of the data collected from the reports) for the consecutive years (2017-2021) was done to assess the progress and success of the NFME. The API showed a statistically significant (ANOVA and chi-square) reduction from 2017-2021 of API in all states/UTs along with category-1 (P=0.003) and category-2 (P=0.0291) states/UTs, but there was no statistically significant reduction from 2017-2021 in category-3 (P=0.1655) states/UTs. The ABER has shown a statistically significant reduction (P=0.04) from 2017 (10.36)-2021 (7.65) in all states/UTs. The AFI did not show a statistically significant reduction (P=0.132) from 2017 (0.65)-2021 (0.225) in all states/UTs. The *Pf%* showed an increase from 2017 (29.81)-2021 (36.71), though non-significant in all states/UTs. The SFR showed a statistically significant reduction (P=0.067) from 2017 (0.76)-2021 (0.141) in all states/UTs. The SPR shows a statistically significant reduction (P=0.011) from 2017(0.75)-2021 (0.210) in all states/UTs.

The number of malaria cases showed statistically significant reduction from 2014 (1102205)-2020 (180423) in all states/UTs(P=0.00907). The same trend continued in category-2 states/UTs from 2014 (23137) to 2020 (66394) (P=0.001612) and category-3 from 2014 (845503) to 2020 (112018) (P=0.027588) states/UTs with statistically significant reduction, whereas there was no statistically significant reduction (P=0.060548) in category-1 states/UTs from 2014 (25305) to 2020 (2011) (Table 2).

**Table 2:** Malaria interrupted and zero indigenous cases and deaths in category-1, category-2, category-3 states/UTs from 2014-2020

State/UTs	Malaria cases		P Value	Deaths		P Value
	
	2014	2020		2014	2020	
Category 1	25305	2011	0.060548	11	4	0.161919
Category 2	23137	66394	**0.001612**	165	23	**0.04354**
Category 3	845503	112018	**0.027588**	386	66	**0.009756**
All states/UTs	1102205	180423	**0.00907**	413	93	**0.002013**

The number of deaths has showed a statistically significant reduction from 2014 to 2020 in all states (P=0.002013). When measuring state-wise, category-2 decreased from 2014 (165) to 2020 (23) (P=0.04354). Category-3 also showed a statistically significant reduction from 2014 (386) to 2020 (66) (P=0.009756). However, there was no statistically significant reduction (P=0.161919) in category-1 states from 2014 (11) to 2020 (4) (Table 2).

Comparison of the epidemiological indicators of individual years from 2017-2021, category state-wise, is depicted in Table 3. The study assessment parameters, i.e. SPR(P=0.1349), SFR(P=0.0965), and *Pf*%(P=0.4103) did not show statistically significant reduction, and ABER did not show a statistically significant change (P=0.1455) in category-1 states/UTs from 2014-2020. There was increase in *Pf* % from 2014 to 2020 in category-2 States/UTs and Category-3 States/UTs; there was no statistically significant reduction (P=0.4103) of *Pf %* from 2014-2020 (Table 3).

**Table 3:** Comparison of epidemiological indicators for the years of 2017-2021

		2017-2018			2018-2019			2019-2020			2020-2021			2014-2020		

		2017 (Mean)	2018 (Mean)	*P value	2018 (Mean)	2019 (Mean)	*P value	2019 (Mean)	2020 (mean)	*P value	2020 (Mean)	2021 (Mean)	*P value	2014 (Mean)	2020 (Mean)	*P value
	API	0.088	0.057	**0.03**	0.057	0.043	0.083	0.043	0.015	**0.003**	0.015	0.009	0.08	0.12	0.015	**0.004**
**Category-1**	SPR	0.129	0.158	0.331	0.158	0.225	0.172	0.225	0.071	0.146	0.071	0.067	0.442	0.145	0.071	0.135
	PF%	10.191	9.772	0.395	9.772	9.418	0.445	9.418	9.422	0.499	9.422	18.219	**0.018**	10.4	9.422	0.41
	SFR	0.009	0.004	**0.007**	0.004	0.004	0.418	0.004	0.005	0.417	0.005	0.012	**0.022**	0.027	0.005	0.097
	ABER	8.034	7.504	**0.012**	7.504	6.96	0.159	6.96	5.751	0.293	5.751	4.073	0.193	8.496	5.751	0.146
	AFI	0.009	0.005	**0.014**	0.005	0.004	0.341	0.004	0.002	**0.033**	0.002	0.003	0.113	0.013	0.002	**0.042**
**Category-2**	API	0.203	0.144	0.106	0.141	0.109	**0.016**	0.109	0.051	**0.029**	0.051	**0.058**	**0.328**	0.361	0.051	**0.002**
	SPR	0.318	0.321	0.489	0.321	0.265	**0.01**	0.265	0.199	0.102	0.199	0.098	0.122	0.436	0.199	**0.007**
	PF%	31.077	32.606	0.363	32.606	29.501	0.049	29.501	43.046	0.016	43.046	37.911	0.173	36.636	43.046	0.112
	SFR	1.037	0.088	0.175	0.088	0.048	**0.021**	0.048	0.063	0.087	0.063	0.032	0.141	0.14	0.063	**0.051**
	ABER	11.11	10.727	**0.037**	10.727	11.603	0.094	11.603	7.331	**0**	7.331	8.771	**0.001**	11.753	7.331	**0.001**
	AFI	0.074	0.038	0.064	0.038	0.022	**0.02**	0.022	0.017	0.172	0.017	0.023	0.216	0.137	0.017	**0.006**
**Category-3**	API	3.187	1.706	**0.04**	1.591	1.583	0.492	1.583	1.11	**0.028**	1.11	0.947	0.291	7.167	1.11	**0.002**
	SPR	2.19	1.269	**0.03**	1.269	0.97	0.133	0.97	0.716	**0.033**	0.716	0.55	0.137	4.446	0.716	**0.001**
	PF%	57.869	54.831	0.105	54.831	57.625	0.139	57.625	72.486	0.083	72.486	63.147	0.163	61.3	72.486	0.132
	SFR	1.607	0.935	**0.055**	0.935	0.794	0.284	0.794	0.066	**0.033**	0.066	0.455	**0.042**	3.513	0.066	**0.005**
	ABER	13.009	12.226	0.09	12.226	14.45	**0.004**	14.45	11.095	**0.003**	11.095	16.589	0.169	15.369	11.095	**0**
	AFI	2.255	1.222	**0.057**	1.222	1.346	0.374	1.346	0.916	**0.04**	0.9161	0.782	0.296	5.944	0.916	**0.008**

**Figure 2:**
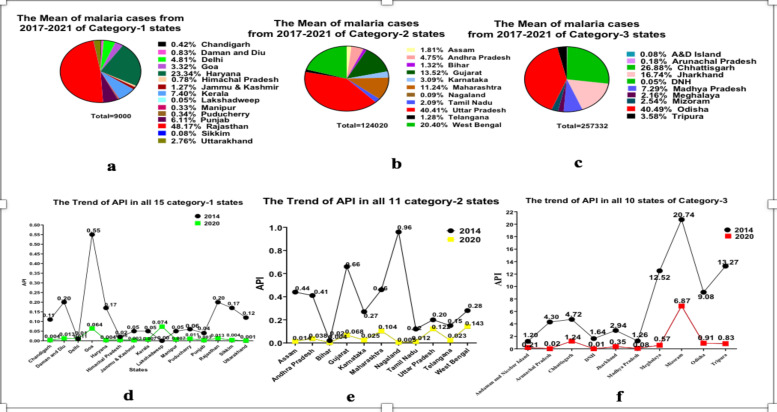
**a)** Mean of malaria cases from 2017-2020 of category-1 states/UTs. **b)** Mean of malaria cases from 2017-2020 of category-2 states/UTs. **c)** Mean of malaria cases from 2017-2020 of category-3 states/UTs **d)** Trend of API in all 15 category-1 states/UTs for 2014 and 2020. **e)** Trend of API in all 11 category-2 states/UTs for 2014 and 2020. **f)** Trend of API in all 10 category-3 states/UTs for 2014 and 2020

**Figure 3:**
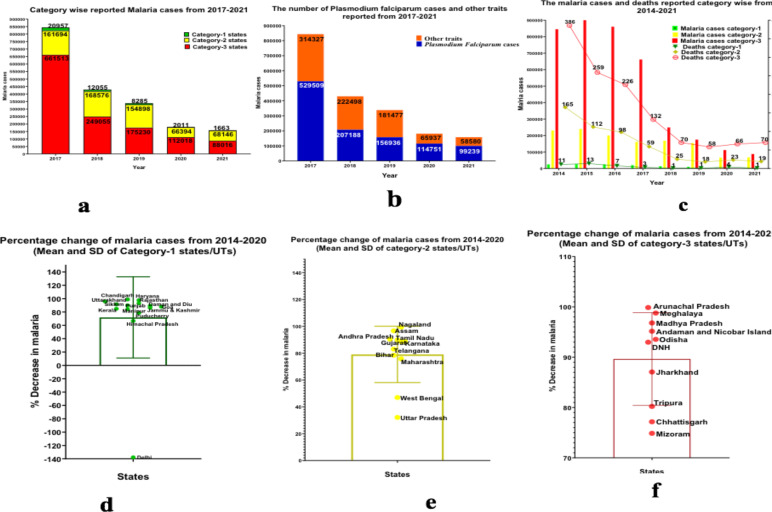
**a)** Category wise reported malaria cases from 2017-2021 **b)** The number of cases and other traits reported from 2017-2021 **c)** The malaria cases and deaths reported category wise from 2014-2021 **d)** The percentage change of malaria from 2014-2020 (Mean and SD) in category-1 states/UTs **e)** The percentage change of malaria from 2014-2020 (Mean and SD) in category-2 states/UTs **f)** The percentage change of plasmodium falciparum malaria from 2014-2020 (Mean and SD) in category-3 states/UTs

The trends of API for the year of 2014 and for 2020 are represented in Figure 2 for all states (category-wise) along with the mean of malaria cases from 2017-2021 in all states (category-wise). The overall percentage reduction in the number of malaria cases by 2020 at the national level compared with 2014 was 83.6%. The percentage reduction in the number of malaria cases by 2020 compared with 2014 in category-1, category-2, and category-3 was 92.0%, 71.3% and 89.5 %, respectively. The mean percentage decrease of malaria cases between the states of category-1 (71.9), category-2 (79.1), and category-3 (89.6) is illustrated in Figure 3 along with the confidence interval. However, But, the two states, Delhi showed an increase in malaria cases from 2014 (98) to 2020 (233) and Lakshadweep from 2014 (0) and 2020 (5). The progressive states with strong health systems such as Gujarat, Maharashtra, and Karnataka did not show zero indigenous cases in 2020, and the malaria cases noted were very far from reaching the targets, such as 4777, 12909 and 1663 malaria cases, respectively.

## Discussion

Based on the reports collected for our study, we noted a significant decline of malaria incidence from 2014-2020, which shows the country to head towards the elimination of malaria.

In category-1 states/UTs, there was a difference between the expected API (P=0.00) and the observed API (P=0.0085). Though these states/UTs failed to reach zero indigenous cases, the difference between them is very close. There was no statistically significant reduction (P=0.16) of deaths from 2014 (11) to 2020 (4).

Our findings revealed that, in category-1 states/UTs, the study assessment parameters i.e., epidemiological indicators like SPR, SFR, *Pf%*, and ABER, did not show any significant change. Hence, there is a need for rigorous surveillance and implementation of new initiatives to reach the targets in category-1 states/UTs. Among the category-1 states/UTs, Rajasthan showed the highest mean number of malaria cases for the years 2017-2021, despite various measures including prevention of breeding places by intervention of larval control in stagnated water sites (13) and replacement of damaged lids on underground water storage tanks (14). The highest number of malaria cases in Rajasthan were due to seasonal variation and low socioeconomic group of people (15).

Though Lakshadweep showed lowest mean number of malaria cases in category-1 states for the years 2017-2021, it showed an increase in the number of cases from 2014. While Sikkim demonstrated the lowest number of malaria cases among category-1 states/UTs from 2017 to 2021, the persistence of indigenous cases can be attributed to factors such as the influx of laborers from malaria-endemic regions for construction and project work, the presence of army personnel, and the local population residing in the lower regions of the state (16).

The overall reduction of API score showed statistically significant(0.02) amongst category-2 states/UTs. However, the data was limited with regard to district-wise API. Hence, it cannot be possible to estimate whether the state can enter into category-1 from category-2. Among the category-2 states/UTs, Uttar Pradesh showed highest mean number of malaria cases for the years 2017-2021 due to many reasons including excessive rainfall, poor surveillance due to inadequate number of peripheral health workers, lack of laboratory facilities and improper monitoring, and lack of timely actions (17). West Bengal showed second highest mean number of malaria cases in category-2 states/UTs from the years 2017-2021 despite, various steps to control vector borne diseases, include updating case management guides and time-to-time polices regarding the vector borne diseases (18). The contributing factors for increase in the number of cases were of water pollution and positive indicator of larval density (19). In their study conducted in Maharashtra, Dr. Sachin Gupta and Dr. Arunesh Kumar highlighted that the third-highest average number of malarial cases in the state could be attributed to factors, such as incomplete fulfilment of expected duties by ASHA workers and lack of cooperation from migratory individuals towards ASHA workers (20).

Our study finding reveals that there was no statistically significant increase in ABER (operational efficacy of the programme) from 2014-2020 in category-1 states/UTs. However, there was a statistically significant decrease was noticed from 2014-2020 in category-2 and category-3 states/UTs. The trend of ABER from 2017-2021 shows a positive correlation with API and SPR. This shows that the decrease in the API has been associated with the decrease in ABER.

The states/UTs in the category-3, Andaman and Nicobar Island, Arunachal Pradesh, DNH, Jharkhand, Madhya Pradesh, Meghalaya, and Odisha had API less than one in 2020.

The district data collected from annual report of 2017 and annual report of 2018 showed that these states fulfilled the criteria for entering into category-2. However, the global pandemic COVID-19 might have had an influence in the decline in the number of malaria cases in the year 2020. This could be due to lower vector transmission as a result of social distancing and lockdown or due to underreporting amidst the COVID-19 pandemic (21).

Among category-3 states/UTs, Odisha showed the highest mean number of malaria cases for the years 2017-2021, due to many reasons, including the wide distribution of *Plasmodium ovale* and *Plasmodium malariae* in different geophysical regions of the state (22).

Chhattisgarh showed the second highest mean number of malaria cases in category-3 states/UTs for the years 2017-2021. Currently, Chhattisgarh has been actively executing a range of measures since 2019, including the “Malaria-Mukt Bastar” initiative, resulting in a significant reduction of approximately 49% in malaria cases following the campaign (23). Jharkhand showed that third highest mean number of malaria cases in category-3 states/UTs for the years 2017-2021, as the increase in the number of malaria cases in this state was associated with the forest area of Jharkhand as tribal villages consist of numerous hills, streams along with their tributaries which maintain mosquito breeding throughout the year and these communities prefer their treatment with untrained practitioner or spiritual healers (24).

Despite 3.6% reduction of malaria cases by 2020 compared with 2014 national wide, the increase in the number of malaria cases in Delhi were bothersome to the policy makers due to rainfall and humidity which were strong predictors of malaria infection (25). Lakshadweep, which showed a five-fold increase in number of malaria cases, was of presence of different vector species in the islands, favourable breeding sites, and water storage practices (26). However, it has showed a non-linear increase in the number of malaria cases from 2014-2021.

The ASHA workers play a crucial role (i.e., diagnosis and treatment of malaria cases) in eliminating malaria, Harsh Rajvanshi et.al, in their study, identified that most of ASHAs reported diagnosis of malaria through RDTs and blood slides, but only 10–15% could recognize *P. vivax* and *P. falciparum* RDTs correctly. ASHA's awareness to ACT colour packs for different age groups was also poor, and PQ doses were not properly administered in the case of *P. vivax* and *P. falciparum* malaria cases. There is a need for fresh and continuous training of ASHAs to achieve the goal of malaria elimination in India (27).

Although the percentage decline in the number of malaria cases in the states with strong health systems, such as Gujarat, Maharashtra, and Karnataka were high, the achievement of zero indigenous cases in 2020 was not reached. Socioeconomic status of the patients, poor education, low income, and living in the poorly constructed house were associated with the risk of infection in Gujarat (28,29). Numerous studies conducted in different countries have also showcased the potential impact of poverty, and potentially, the socio-economic status of the population in the endeavours to control and alleviate the burden of malaria (30). While in Maharashtra, the reasons include gaps in knowledge on malaria at a community level, ignorance and treatment by unqualified traditional healers, delay in effective treatment (31), and poor performance of the ASHA workers, and there is a need for continuing focus on these vulnerable sections (32). In Karnataka, the awareness about malaria and prevention was low (33). Hence, there is a strong urge for development of new strategies such as gene drive, bacillus thuringiensis israelensis, attractive toxic sugar bait (ATSB), sterile insect technique, optical approaches, and their effective implementation for malaria elimination (34).

The public-private partnerships have proven to be beneficial in controlling malaria in high-burden districts of India; the two successful ones are the Comprehensive Case Management Project (CCMP) in Odisha, which was adopted by the state, and the Malaria Elimination Demonstration Project (MEDP), which has nearly eliminated malaria from the highly endemic district of Mandla in Madhya Pradesh (35).

There was no statistically significant reduction of the *Pf%* in the country from 2014 to 2018, suggesting the need to analyse the burden of individual species and to develop and implement new strategies to reduce *Pf%* (36).

The drug resistance found in various regions in India should be taken into into consideration while framing new strategies (37,38). This study's outcomes involve a comprehensive analysis of the progression and resultant effects of the established framework. Moreover, as previously indicated, the requisite training of ASHA workers and staff is imperative. This is particularly crucial since inadequate staff performance has been identified as a primary factor in the increase of malaria incidences within numerous states. Various studies from different countries have also demonstrated that providing training on malaria has enhanced the understanding of malaria prevention and control among role model community caregivers, contributing to the effective execution of malaria control Programs (39).

The barriers responsible for the increase of malaria cases was different from each geographical area. Hence, we recommend effective preventive measures of malaria and strengthen rapid investigation techniques to early diagnosis remove malaria in malaria endemic areas. The NVBDCP has started publishing monthly reports only from 2020, and there is no such data on monthly reports were available for the years 2017,2018 and 2019. Hence, this study lacks the analysis of monthly reports of malaria.

Although we observed a significant drop in malaria incidence from 2014 to 2020, demonstrating that the country is moving nearer to malaria elimination, it is crucial to implement the strategies to reduce *Plasmodium falciparum%* as there is no statistically significant reduction in *Pf%* from 2014 to 2020 in all states/UTs. In fact, an increase in *Pf%* was observed in category-2 and category -3 states. The global pandemic COVID-19 might have contributed to underreporting of malaria cases in 2020. There was a statistically significant reduction of SPR and SFR from 2017-2021, and the same happened throughout the study assessment years, which implies that India is stepping towards meeting its milestone of NFME. Our results imply that there is an urgent need to re-establish surveillance programmes and execute national and state programmes in order to achieve the success of the National Malaria Elimination Programme. The recategorization of states/UTs and implementation of effective strategies in accordance with API in poor performing states/UTs is highly recommended.

## Figures and Tables

**Table 1:** API from 2017-2020 for all states including category-1, category-2 and category-3

Variables	ANOVA		Chi-square test	

	F	P value	χ^2^	P value
API for all States (2017-2020)	21.3827	0.00	387.203	0.00
API of cat-1 States (2017-2020)	4.3311	0.003	55.4918	0.00
API of cat-2 States (2017-2020)	2.94302	0.0291	12.8072	0.0123
API of cat-3 States (2017-2020)	1.70486	0.1655	5.02742	0.284
